# The Feasibility of Practical Training in Minimally Invasive Surgery at Medical School—A Prospective Study on the Pelvitrainer

**DOI:** 10.3390/medicina60010159

**Published:** 2024-01-15

**Authors:** Johannes Ackermann, Julian Pape, Felix Vogler, Julia Pahls, Jorun Baumann, Bernd Holthaus, Günter Karl Noé, Michael Anapolski, Zino Ruchay, Anna Westermann, Veronika Günther, Kristin Andresen, Leila Allahqoli, Gaby Moawad, Jörg Neymeyer, Sandra Brügge, Nicolai Maass, Liselotte Mettler, Ibrahim Alkatout

**Affiliations:** 1 Kiel School of Gynaecological Endoscopy, Department of Obstetrics and Gynecology, University Hospitals Schleswig-Holstein, Campus Kiel, Arnold-Heller-Str. 3 (House C), 24105 Kiel, Germany; julianmaria.pape@uksh.de (J.P.); felixvogler@t-online.de (F.V.); julia.pahls@uksh.de (J.P.); jorun.baumann@uksh.de (J.B.); annamaria.westermann@uksh.de (A.W.); veronika.guenther@uksh.de (V.G.); kristin.andresen@uksh.de (K.A.); sandra.bruegge@uksh.de (S.B.); nicolai.maass@uksh.de (N.M.); profmettler@gmx.de (L.M.); 2 Clinic of Obstetrics and Gynecology, St. Elisabeth Hospital, 49401 Damme, Germany; b.holthaus@t-online.de; 3 Department of Obstetrics and Gynecology, University Witten/Herdecke, Rheinland Klinikum Dormagen, Dr.-Geldmacher-Straße 20, 41540 Dormagen, Germany; karl-guenter.noe@kkh-ne.de (G.K.N.);; 4 Midwifery Department, Ministry of Health and Medical Education, Tehran 1467664961, Iran; lallahqoli@gmail.com; 5 Department of Obstetrics and Gynaecology, The George Washington University Hospital, Washington, DC 20037, USA; gnmoawad@gmail.com; 6 Clinic of Urology, Charité—Universitätsmedizin Berlin, Hindenburgdamm 30, 12200 Berlin, Germany; joerg.neymeyer@charite.de

**Keywords:** medical student education, practical surgical training, pelvitrainer, laparoscopic surgery

## Abstract

*Background and Objectives*: The acquisition of practical skills at medical school is an important part of the multidimensional education program of future physicians. However, medical schools throughout the world have been slow in incorporating practical skills in their curriculum. Therefore, the aims of the present prospective study were (a) to demonstrate the feasibility of such surgical training, (b) to objectify its benefit in medical education, and (c) to investigate the impact of such training on subsequent career choices. *Material and Methods:* We introduced a two-day laparoscopy course on the pelvitrainer as part of the curriculum of the gynecological internship of fifth year medical students from 2019 to 2020. The results of the students’ training were matched to those of surgeons who completed the same curriculum in a professional postgraduate laparoscopy course from 2017 to 2020 in a comparative study design. Additionally, we performed a questionnaire-based evaluation of the impact of the course on medical education and subsequent career choices directly before and after completing the course. *Results:* A total of 261 medical students and 206 physicians completed the training program. At baseline, the students performed significantly more poorly than physicians in a median of three of four exercises (*p* < 0.001). However, this evened out in the final runs, during which students performed more poorly than physicians only in one exercise and even better than physicians in one. The general integration of surgical training in medical school curricula was rated very low (12.4% on the VAS, IQR 3–16%) despite the high demand for such training. In the survey, the course was deemed very beneficial for medical education (median VAS 80.7%, IQR 73–98%), but did not appear to influence the students’ subsequent career preferences. *Conclusions:* The acquisition of practical surgical skills during medical school is significantly under-represented in many medical faculties. The benefits of such training, as demonstrated in our study, would improve the education of future physicians.

## 1. Introduction

The acquisition of practical skills during medical school is an important aspect of the education of future physicians [1,2]. However, the incorporation of such skills in medical education has been slow throughout the world [3]. For surgical specialties in particular, it appears even more difficult to integrate practical skills into medical education [4]. While the establishment of skills labs is now standard practice at many universities, the actual training frequently does not go beyond practicing suturing and knotting techniques [5]. In view of the fact that the early acquisition of practical skills in surgery is also associated with the earlier recruitment of talented students and with a steeper learning curve that will help future surgeons, a change in this sector may be considered essential [6]. Furthermore, introducing medical students to the practical aspects of surgery early will assist them in making career choices in later life [7]. This is particularly important in view of the increasing shortage of resources in health care systems.

Minimally invasive surgery has become the gold standard for many surgical indications. However, minimally invasive techniques require special skills in terms of spatial awareness and manual dexterity [8,9]. The keyhole approach is associated with a flatter and longer learning curve than those for other medical interventions [10].

However, minimally invasive surgery offers a wide range of high-quality training options away from the patient [11]. As early as in the 1980s, Kurt Semm developed the pelvitrainer [12,13,14]. The latter is a simple, low-cost, and very effective training device for acquiring laparoscopic skills and still is the most widely used training device for laparoscopic surgery worldwide [15]. Its widespread availability, ease of use, and low cost make it an ideal training tool for aspiring surgeons and students [16].

The pelvitrainer has been used at our clinic for several years in postgraduate medical training as well as medical school. The subjective benefit of this training is rated high by the students. The aims of the present prospective study were (a) to demonstrate the feasibility of such surgical training, (b) to objectify its benefit in medical education when used in a structured training program, and (c) to investigate the impact of such training on subsequent career choices. To the best of our knowledge, the present work is the first to demonstrate, in a prospective setting, the actual benefit of a structured training program on the pelvitrainer at medical school.

## 2. Materials and Methods

As part of their gynecological internship of medical school curriculum, students in their fifth year (last year of medical school before the final year clinical elective) at Christian Albrechts University in Kiel completed a two-day laparoscopy course on the pelvitrainer in 2019–2020 (two semesters: winter 2019 and summer 2020). The exercises were performed at the Kiel School of Gynecological Endoscopy, University Hospital of Schleswig-Holstein, in Kiel. The Kiel School of Gynecological Endoscopy is a training center for minimally invasive surgery certified by the German Gynecological Endoscopy Working Group (AGE e.V.) and affiliated to the Department of Obstetrics and Gynecology. Physicians from 16 standardized training courses of the German Gynecological Endoscopy Working Group (AGE e.V.) at the three sites in Kiel, Damme, and Dormagen, who had undergone the same training program from 2017 to 2020, were used as a control group to compare the training outcomes of the students. The study was approved by the ethics committee of the Medical Faculty of Kiel University on the 20th of June 2017 (approval number D 484/17).

**Figure 1:**
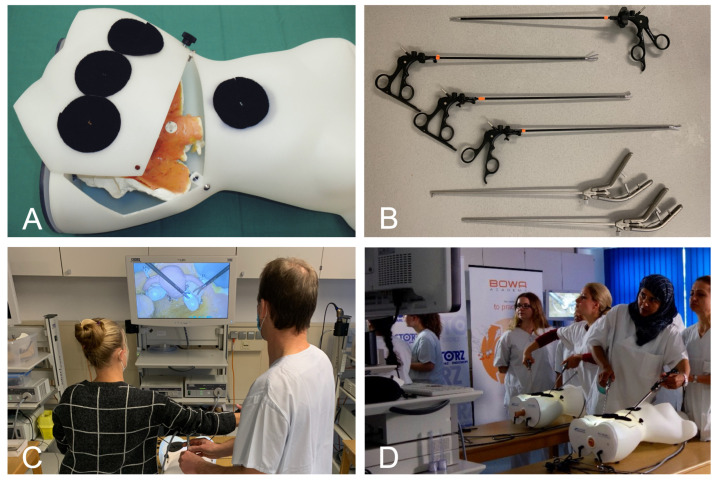
Training setup. (**A**). The pelvitrainer Realsimulator 2.0. (**B**). Laparoscopic instruments. (**C**). Laparoscopic setup and imaging system. (**D**). Attendees of the training course. From Ackermann et al., 2023 [6].

The training program on the pelvitrainer, completed by the students, was the same as a standardized training course designed earlier for gynecologists, general surgeons, and urologists. The data collection for the physicians was also performed in a prospective setting. The training concept, results, and learning curves of physicians were published earlier by the working group [15]. The laparoscopic exercises were carried out on the pelvitrainer Realsimulator 2.0 (Endodevelop, Pelvic School Saarbrücken, Hohe Wacht 77, 66,119 Saarbrücken, Germany). The Realsimulator 2.0 represents an evolution of the classic pelvitrainer. Due to the shape based on the female anatomy, a realistic trocar placement with realistic anatomical relationships in the female pelvis is achieved. Special interchangeable pelvic inserts allow the simulation of complex surgical steps. Due to the used materials, a realistic tactile feedback is achieved [17]. For laparoscopic equipment, complete and real surgical equipment from the company Karl Storz SE & Co. KG (Dr.-Karl-Storz-Straße 34, 78,532 Tuttlingen, Germany) was available. In Figure 1, the pelvitrainer and equipment are shown.

Two students shared a training station. The two students alternated as camera assistant and surgeon for each exercise. A total of 5 training devices (pelvitrainer and laparoscopic equipment) were available; therefore, the number of course participants was limited to 10. The training took place on two consecutive days of the two-week gynecological internship. Due to the limited group size, several courses were held during these two weeks to ensure that all students were able to take part in the training. The training was supervised by a teaching consultant, and the individual stations were supervised by two trained doctoral students (J.P. and F.V.) who measured and checked the respective exercises.

**Figure 2:**
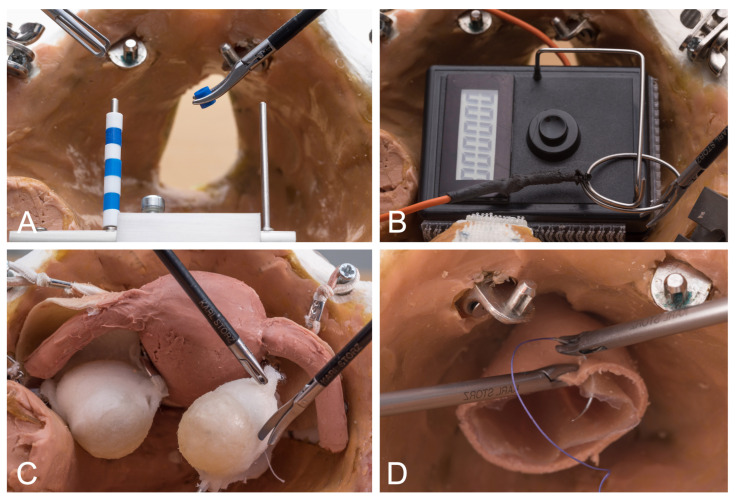
Laparoscopic exercises. (**A**). Moving Pearls. (**B**). Hot Wire. (**C**). Vaginal Vault Closure. (**D**). Ovarian Cyst Enucleation. From Ackermann et al., 2023 [6].

The training program consisted of four exercises of increasing difficulty, which had to be repeated several times by the participants. In the first exercise (Moving Pearls, three repetitions, Figure 2A), a total of 8 pearls are moved from one metal rod to another and back again using two surgical gripping instruments. At the end, the success of the exercise is measured by time/pearl. In the second exercise (Hot Wire, three repetitions, Figure 2B), a metal ring is passed over a metal wire without touching it. Touches are recorded digitally. At the end, the success of the exercise is measured in time/contact. In the third exercise (Vaginal Vault Closure, two repetitions, Figure 2C), two intracorporeal laparoscopic knots are to be tied on an artificial vaginal stump after hysterectomy. Success is measured quantitatively with time/knot and qualitatively with the OSAT score [18]. In the fourth exercise (Ovarian Cyst Enucleation, two repetitions, Figure 2D), an ovarian cyst has to be enucleated from a realistic ovarian model without rupturing the cyst. Success is measured by the time required for enucleation without rupturing the cyst.

To determine the value of the laparoscopy training program for medical education, we conducted a questionnaire-based survey on the same day before and after the students’ attendance of the course. The questionnaire was developed with a statistician. The survey objectives were to assess the following aspects: (a) the impact of the course on subsequent career choices, (b) the usefulness of the course in supporting later career choices, (c) the usefulness of the course in medical training, and (d) the need to acquire surgical skills during medical school. The students’ evaluation was registered on a visual analog scale (VAS). The questionnaire was approved by a medical statistician.

Participation in the training program was mandatory for all students. However, the training results were not included in the final grade for the internship. Participation in the study was voluntary. Written consent was obtained from each participant.

The IBM SPSS Statistics 23 program was used for statistical analysis (IBM, Armonk, NY, USA). Qualitative variables were described by frequency (percentage), and quantitative variables were presented descriptively as means and standard deviations, minimum, maximum, quartiles, and interquartile ranges (IQR). The one-sample Kolmogorov–Smirnov test was used to test the normal distribution of quantitative data. The Mann–Whitney U test was used for subgroup analysis of nonparametric data, or the Kruskal–Wallis test for more than two subgroups. Tests were performed bilaterally and the level of significance was set to 5% (*p* < 0.05).

## 3. Results

A total of 261 fifth-year medical students attended the structured training program as part of their clinical clerkship in gynecology. Previously, 206 physicians from 21 countries had undergone the training program and were used as a reference group to evaluate the results of the course in students. Of the 206 physicians, 180 (success rate/response rate 87.4%) had completed the multi-stage training program. Table 1 provides a summary of sociodemographic data and differences between medical students and physicians. There was a significant difference in age (median 25 years vs. 36 years, *p* < 0.001) and handedness (right hand: 92.8% vs. 98.9%, *p* < 0.05) between the two groups. These data have been published in part elsewhere [6].

**Figure 3:**
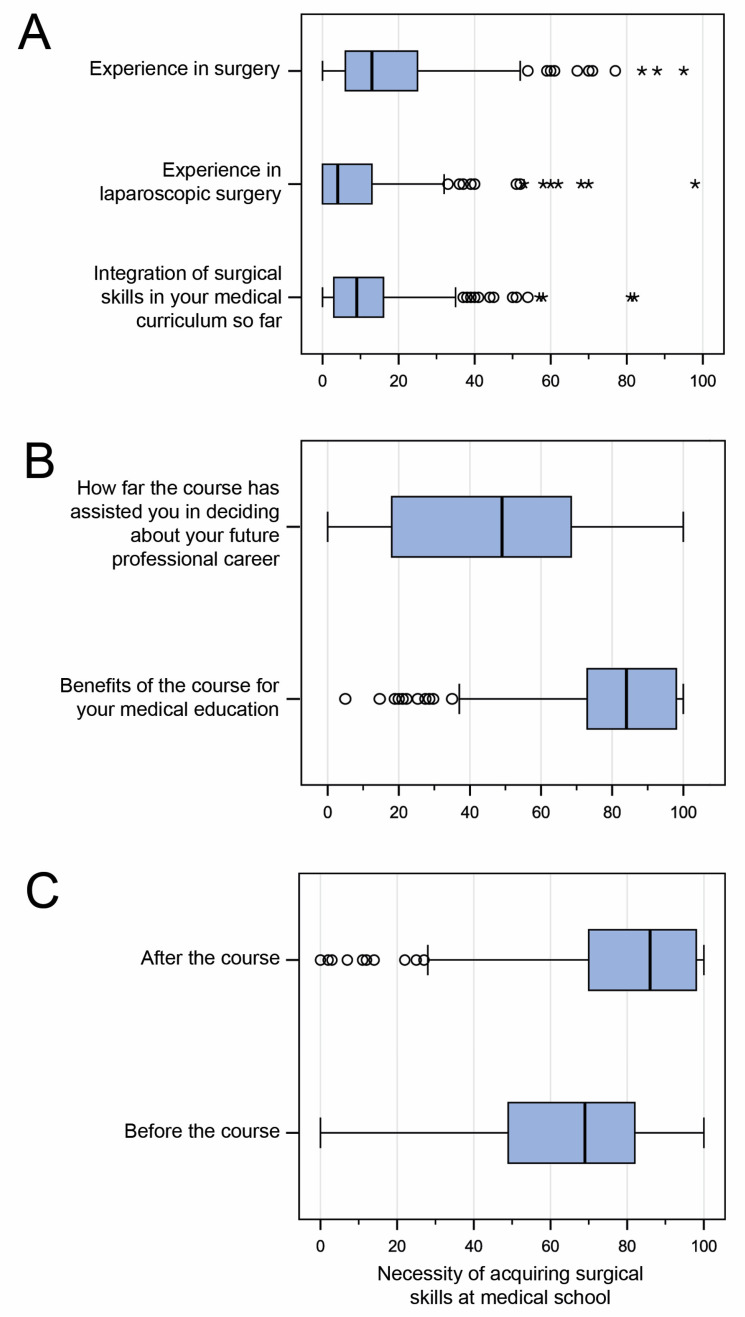
Evaluation of the training program by the students. (**A**). Previous practical experience in surgery, previous experience in laparoscopic surgery, and previous integration of surgical skills training into medical studies. (**B**). Value of the course in influencing future career choices and its benefits for medical education. (**C**). Assessment of the need to acquire surgical skills at medical school: before and after the course.

The survey among students in regard to prior surgical experience yielded very low overall scores: 50.2% (131/261) reported prior experience in a surgical specialty. A mere 31% (81/261) had prior experience in minimally invasive surgery. On the visual analog scale, the students’ self-assessment of surgical skills was given a median rating of 18.3% (IQR 6–25%), while prior experience in minimally invasive surgery was given a low rating of 9.7% (IQR 0–13.5%). Previous integration of hands-on surgical skills in the medical curriculum was given a median rating of 12.4% on the VAS (IQR 3–16%). See Figure 3.

**Figure 4:**
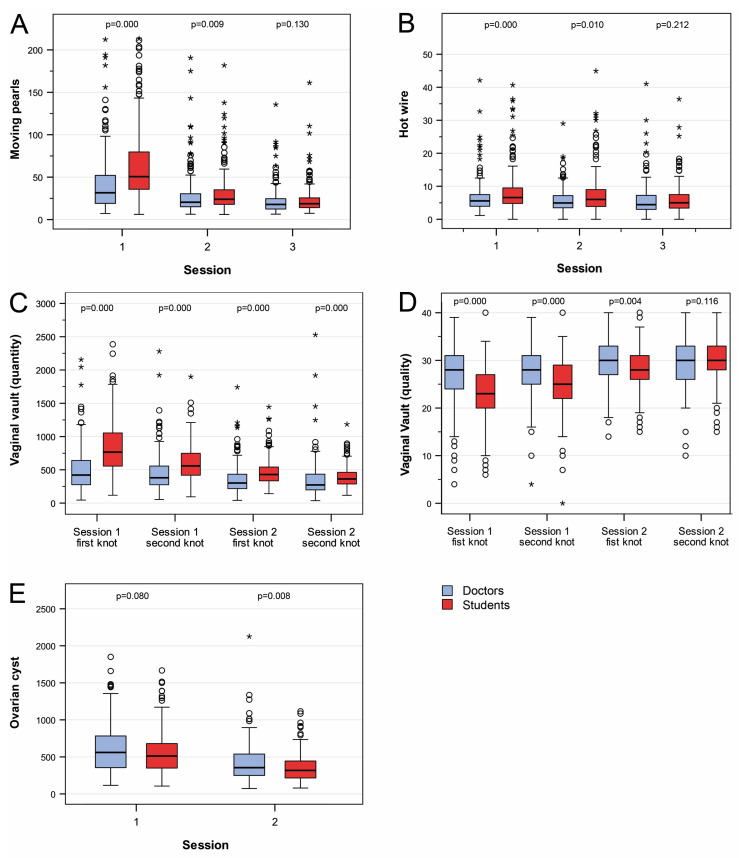
Results achieved by physicians and students. (**A**). Moving Pearls. (**B**). Hot Wire. (**C**). Vaginal Vault (quantity). (**D**). Vaginal Vault (quality). (**E**). Ovarian Cyst Enucleation.

Analogous to physicians, the skills of students improved significantly for all exercises (*p* < 0.001). Initially, three of the four exercises (Moving Pearls, Hot Wire, and Vaginal Vault Closure) yielded significantly poorer results in students compared to physicians (*p* < 0.001). However, this evened out for the basic Moving Pearls and Hot Wire exercises after three practice sessions (Moving Pearls: mean 22.5 s/pearl (SD 17.6 s/pearl) vs. 22.8 s/pearl (SD 16.2 s/pearl), *p* = 0.13; Hot Wire: mean 6.0 s/contact (SD 5.3 s/contact) vs. 5.9 s/contact (SD 4.0 s/contact); *p* = 0.21; Figure 4A,B). In the more complex suturing exercise of vaginal closure, a similarity was noted in knotting quality (OSAT score) at the fourth knot (mean OSAT: 30 points vs. 30 points, *p* = 0.116; Figure 4C). Only in terms of the time taken per knot, students were unable to reach the level of physicians. However, a convergence of time was noted for this exercise as well (mean seconds/knot: 363 s/knot vs. 273 s/knot; *p* < 0.001; Figure 4D). In the complex dissection exercise Ovarian Cyst Enucleation, students were significantly faster in the second run (mean time: 317 s vs. 355 s, *p* < 0.01). Additionally, there were significantly fewer cyst ruptures among students (number of cyst ruptures 4.7% vs. 21.2%; *p* < 0.001; Figure 4E). In the group of students, handedness had no influence on the training result. Due to the lack of statistical power, we were unable to address the significant difference in handedness between doctors and students.

**Figure 5:**
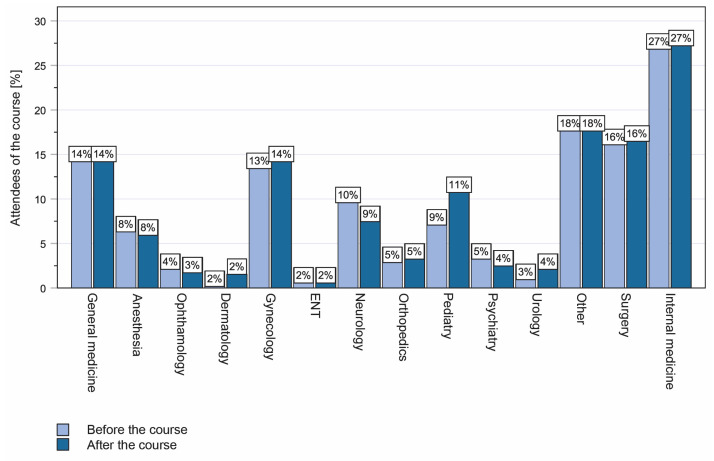
Students’ career aspirations before and after the training course with regard to their preferred clinical specialty. No significant differences were seen.

In order to determine the impact of the course on subsequent professional choices, we conducted a survey before and after the course in regard to career aspirations (Figure 5). The survey did not reveal any significant changes. The observation was confirmed when the students were asked whether the laparoscopy course had helped them in making decisions about their future career. The median benefit reported here was 46.4% (IQR 18–61%) on the VAS (Figure 3B). In contrast, medical students rated the course very highly in terms of its usefulness for their medical education (median VAS 80.7%, IQR 73–98%, Figure 3B). Interestingly, students rated the need for acquiring surgical skills at medical school significantly lower (median) at the beginning of the laparoscopy course than they did after completing the course (median VAS 64.3%, IQR 49–82% vs. 79.4% IQR 70–98%; *p* < 0.001; Figure 3C).

## 4. Discussion

We evaluated an innovative training concept for medical students on the pelvitrainer. The surgical training course was integrated into the curriculum of medical students during their clerkship in gynecology. The multi-stage training concept, originally designed for physicians, was completed by all participating students. The training results of students were comparable to those of physicians, with the difference that students had a significantly steeper learning curve. The value of the course was rated very highly by the students. Yet, it had no impact on their subsequent career choices.

The multilevel training concept, which had been tested and rated positively by physicians working in the field of laparoscopic surgery, served as a template for permanent use in undergraduate training [15]. All of our students in their fifth year of medical school completed the training course in laparoscopy as part of their gynecology clerkship. This demonstrates the feasibility of the educational concept for hands-on training in minimally invasive surgery during medical school. After its successful implementation, the course became an integral part of the medical clerkship in obstetrics and gynecology and could even be conducted during the COVID pandemic except for the initial period of prohibited personal attendance.

Apart from feasibility, we evaluated the benefit of a laparoscopy training course as part of the medical curriculum. The benefit of the course was rated highly by students. The demand for permanent incorporation of surgical skills in undergraduate education was clearly endorsed. The different assessments of the course before and after its implementation were notable: the need to acquire surgical skills during medical school was considered significantly more important after the course than it was prior to the course. On the one hand, this confirms the value of the training program. On the other hand, it shows that the students themselves are unaware of the benefits of practical training. Therefore, it is incumbent upon teachers to offer high-quality education and prepare future physicians optimally for their future tasks [19].

Besides the positive subjective rating of the course by the students, we noted a significant objective training effect. This was more pronounced among students than among physicians. Initially, the students achieved significantly poorer training results, which, however, rapidly reached the performance of physicians due to the significantly steeper learning curve. We were unable to establish how the steeper learning curve actually came about, because we performed no randomization with regard to this question. However, a multiple factor analysis of the attendees revealed the impact of age on training performance and the learning curve [20]. Given the significantly lower age of students, a correlation between a steeper learning curve and younger age would appear quite plausible. Further work focusing on age will provide clarity in this regard. Nevertheless, the observation supports a call for imparting and acquiring surgical skills at medical school. The urgency of this innovation is heightened by the alarmingly low level of practical experience in surgery among medical students, as demonstrated in our survey, and the paucity of such training courses in medical schools.

One aim of our study was to investigate the impact of such training on subsequent career aspirations [21]. We registered no such impact. However, the following points are worthy of note. First, the survey was conducted directly before and after the training. A change of opinion that might have occurred later was not recorded. Second, the course was held during the fifth year of medical school. At such a late stage, the student will have established his/her line of work in most cases, and a change of preferences is unlikely. Third, laparoscopic training is a very specialized subspecialty of surgery and accounts for a small number of surgeries performed. Moreover, surgical specialties comprise many aspects apart from physical skills. Thus, we recommend a more versatile curriculum for the acquisition of surgical skills throughout medical school. Suturing and knotting exercises could be started at an early stage. Clinical and surgical anatomy could be taught by appropriate specialists during dissection courses on body donors. Endoscopy might be offered in the respective specialties, such as general surgery, urology, gynecology, and orthopedics. Synergistic effects would also create a positive impact on postgraduate training. In addition, it would be interesting to find out whether the later career aspirations have an influence on the surgical outcome of the training, i.e., whether the talented students later choose the right discipline. A further study on this issue is currently being conducted by our working group.

## 5. Conclusions

Following the present study, laparoscopy training on the pelvitrainer wasestablished as an integral part of the medical curriculum in our faculty. The ongoing positive evaluations of students also support the maintenance of the course in the future. We were able to show that it is possible to implement a concept of this nature sustainably and permanently. We believe that such training can be integrated into medical curricula in most parts of developed countries. A number of economical and practical training models can be created without major hurdles. The multilevel concept presented here could be easily implemented. Modern media permit exchange and comparisons between students and teachers. We envisage the use of this training course as a model for other universities. The integration of such programs in medical curricula will expand and intensify the scope of medical education as well as improving the skills of future surgeons.

## Figures and Tables

**Table 1:** Description of the attendees (adapted from Ackermann et al. 2023 [6]).

	Medical Students	Physicians	
Number of filled questionnaires (attendees, response rate)	261 (261, 100%)	180 (206, 87.4%)	*p* < 0.001
Age (median)	25 years (range 21–46 years)	36 years (range 26–78 years)	*p* < 0.001
Gender			n.s.
Female	168 (64.4%)	126 (70.0%)	
Male	93 (35.6%)	54 (30.0%)	
Handedness			*p* < 0.05
Right	245 (92.8%)	178 (98.9%)	
Left	16 (6.1%)	2 (1.1%)	
Both	3 (1.1%)	0	
Specialization			
Gynecology	n/a	165 (91.7%)	n/a
Urology	n/a	4 (2.2%)	n/a
General surgery	n/a	11 (6.1%)	n/a
Professional experience (median)	n/a	6.75 years (range 0–34 years)	n/a
Professional status			
Resident	n/a	80 (44.4%)	n/a
Specialist	n/a	72 (40.0%)	n/a
Consultant	n/a	25 (13.9%)	n/a
not specified	n/a	3 (1.7%)	n/a
Number of laparoscopic surgeries as a surgeon (median)	n/a	25 (range 0–5000)	n/a
Number of laparoscopic surgeries as an assistant (median)	0 (range 0–30)	60 (range 0–3000)	*p* < 0.001

## Data Availability

In order to minimize the possibility of unintentionally sharing information that could be used to re-identify private information, a subset of the data generated for the study can be provided by the corresponding author after written contact via e-mail to kiel.school@uksh.de.
